# Dyslipidemia and Retinal Microvascular Health in Children and Adolescents: A Systematic Review

**DOI:** 10.3390/children13060824

**Published:** 2026-06-17

**Authors:** Krenar Xhafa, Urh Groselj

**Affiliations:** 1 Faculty of Medicine, University of Ljubljana, 1000 Ljubljana, Slovenia; kx92753@student.uni-lj.si; 2 Department of Endocrinology, Diabetes and Metabolic Diseases, University Children’s Hospital, University Medical Centre Ljubljana, 1000 Ljubljana, Slovenia; 3 Center for Rare Disease, University Children’s Hospital, University Medical Centre Ljubljana, 1000 Ljubljana, Slovenia

**Keywords:** body composition, dyslipidemia, retinal vessels, child, familial hypercholesterolemia, optical coherence tomography angiography

## Abstract

**Highlights:**

**What are the main findings?**

- Elevated triglycerides, LDL-C, total cholesterol, and apolipoprotein B levels were associated with narrower retinal arteriolar calibers in children and adolescents.
- Familial hypercholesterolemia was associated with reduced retinal capillary density.

**What is the implication of the main finding?**

- Retinal imaging may serve as a non-invasive biomarker for early microvascular dysfunction in pediatric dyslipidemia.
- Early identification and management of dyslipidemia in youth may help reduce future cardiovascular and microvascular complications.

**Abstract:**

**Background/Objectives**: Dyslipidemia is increasingly linked to retinal microvascular changes, yet its impact in pediatric populations remains unclear. Given the retina’s role as a microvascular model, this review evaluates the association between dyslipidemia and retinal vascular alterations in children and adolescents. **Methods**: We conducted a systematic review following the Preferred Reporting Items for Systematic Reviews and Meta-analysis (PRISMA) guidelines to evaluate the association between lipid profiles, body composition, and retinal vascular parameters in individuals younger than 19 years. Four eligible studies were found. **Results**: Triglyceride (TG) levels, total cholesterol (TC) levels, low-density lipoprotein cholesterol (LDL-C), and apolipoprotein B (apoB) levels were associated with smaller retinal arteriole diameters (CRAE), whereas there was no consistent association between venular diameter (CRVE) and lipid levels. In addition, families with familial hypercholesterolemia (FH) had lower capillary densities and larger foveal avascular zone areas detected by optical coherence tomography angiography (OCT-A) compared to control subjects. The presence of both obesity and dyslipidemia resulted in greater degrees of arteriolar narrowing. **Conclusions**: These findings suggest that adverse metabolic profiles in childhood may be linked to early microvascular alterations. However, the limited number of heterogeneous studies highlights the need for larger longitudinal investigations.

## 1. Introduction

Dyslipidemia, a prevalent metabolic condition that is related to cardiovascular disease [1], is becoming increasingly recognized for its effects on the microcirculation and microvascular aspects of the retinal circulation, the retina being an accessible “window” into the systemic microvasculature. Although the macrovascular consequences of dyslipidemia are established, there is very little evidence on the retinal microvascular implications of dyslipidemia, especially in children and adolescents [2,3,4].

Familial hypercholesterolemia (FH), a severe inherited type of dyslipidemia characterized by exceptionally high levels of low-density lipoprotein cholesterol (LDL-C) [5], acts as an example of this risk, in that it has been linked to early systemic endothelial dysfunction and microvascular impairment in various perivascular tissues (e.g., heart, brain and kidneys) [6,7]. As there are structural and functional similarities between the retina and these microvascular beds, the retinal vasculature may be beneficial as a non-invasive indicator of early metabolic dysfunction [8,9,10].

The use of recent advances in retinal imaging, such as optical coherence tomography angiography (OCT-A), allows for evaluation of the microvascular perfusion [11,12]. However, central retinal arteriolar equivalent (CRAE) and central retinal venular equivalent (CRVE) are derived from fundus photographs, not OCT-A. CRAE/CRVE is a measure of vascular caliber and was developed by measuring the diameter of the major arterioles and venules in a standardized zone around the optic disk on color fundus photographs [13] and therefore represents measures of vascular caliber. OCT-A parameters (for example, foveal avascular zone (FAZ) and vessel density) measure microvascular perfusion. However, there is still a limited understanding of dyslipidemia in conjunction with the retinal microvasculature in children.

The aim of this systematic review was to evaluate the association between dyslipidemia and retinal microvascular alterations in children and adolescents. Specifically, we assessed the relationship between lipid abnormalities and retinal vascular parameters, explored potential sex-related differences, and examined whether the coexistence of obesity and dyslipidemia was associated with more pronounced retinal microvascular changes.

## 2. Materials and Methods

### 2.1. Study Design and Search Strategy

Following the PRISMA 2020 guidelines [14], this systematic review aimed to achieve transparency and methodological rigor. This systematic review was conducted and reported in accordance with the PRISMA 2020 statement [14]. The PRISMA 2020 [14] checklist and flow diagram are provided in the Supplementary Materials. The review protocol was not registered in PROSPERO. Retinal microvascular outcomes included retinal vascular and microvascular parameters reported in the eligible studies.

We conducted a comprehensive search of PubMed, Embase, and the Cochrane Library, using keywords related to dyslipidemia and retinal microvascular changes. The following keywords were used: “body composition,” “dyslipidemia,” “retinal vessels,” “child,” “familial hypercholesterolemia”, “OCT-A” in various combinations with the use of Boolean operators.

We also conducted backward and forward citation tracking to discover further sources to include. The search was limited to articles published until December 2024. We used Zotero (v6.0.36) to manage the results, and we removed duplicates manually. The full electronic search strategy for all databases is provided in Supplementary Table S1.

### 2.2. Study Selection Criteria

Studies were selected based on predefined eligibility criteria (Table 1).

**Figure 1:**
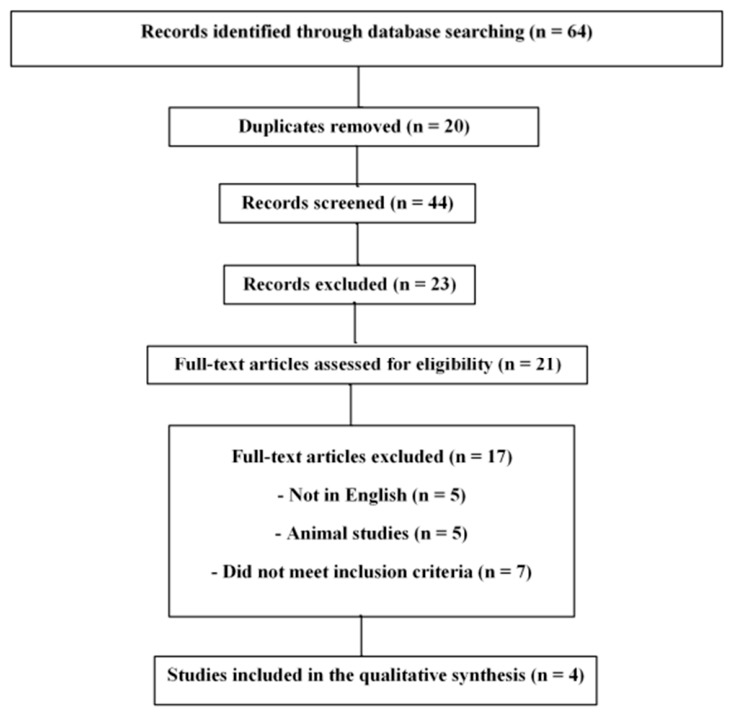
PRISMA flow diagram for study selection.

The studies included were randomized controlled trials (RCTs), cohort studies, case–control studies, and cross-sectional studies of children and adolescents that reported both lipid profiles and body composition (e.g., body mass index (BMI), bioelectrical impedance analysis (BIA), or DEXA). Non-English studies, animal models, reviews, editorials, and case studies were not included. Figure 1 summarizes the search process. In the database searches, a total of 64 records were identified; no records were identified in the registers. After removing 20 duplicate records, 44 unique records remained for screening. After title and abstract screening, 23 records were subsequently excluded, leaving 21 full-text articles for eligibility assessment. In total, 17 articles from these 21 full-text articles were excluded for the following reasons: 5 were not published in English, 5 involved animal models, and 7 did not meet the inclusion criteria. Ultimately, four studies were found to meet eligibility and were included in the systematic review [15,16,17,18].

### 2.3. Characteristics of Included Studies

The primary features of the reviewed studies are listed in Table 2. The total number of studies that were reviewed consisted of three cross-sectional and one case–control study; all of these studies included pediatric participants who resided in China and/or Europe. Two studies primarily assessed lipid–CRAE/CRVE associations [17] (and partially [16]), one focused on body composition [15], and one evaluated FH using OCT-A metrics [18].

### 2.4. Data Extraction and Quality Assessment

Reviewers used a standard form to collect information from all the included studies about the design of the study, demographics of participants, lipid and body fat levels, retinal microvascular outcomes, and quality of each study. The two reviewers collected data for each study individually and assessed quality individually as well, with disagreements resolved by consensus.

The risk of bias of the individual studies that included only observational data was assessed based on the Newcastle–Ottawa Scale (NOS) and scored as follows: all studies that had a NOS score of ≥7 points were deemed to be of high quality, while those with a NOS score of 5–6 points were deemed to be of moderate quality. More detailed assessments are available in Supplementary Table S2. Since there were no randomized controlled trials, it was not possible to assess them using the Cochrane Risk of Bias tool.

### 2.5. Data Synthesis and Analysis

A structured narrative synthesis summarizing the results by retinal vascular measures (CRAE, CRVE), lipid measures (triglycerides (TG), low-density lipoprotein cholesterol (LDL-C), total cholesterol (TC), and high-density lipoprotein cholesterol (HDL-C)) for children and adolescents was conducted because a meta-analysis could not be completed due to the low number of eligible studies, methodological variation among studies and differences in study designs, study populations, and how outcomes were reported.

Retinal vascular measurements (CRAE, CRVE) for all studies were measured via fundus photography; however, OCT-A measurements (superficial capillary plexus (SCP), deep capillary plexus (DCP), FAZ, and radial peripapillary capillary (RPC)) were only available for one of the studies reviewed.

## 3. Results

### 3.1. Dyslipidemia and Retinal Vascular Health

TG levels had a direct negative effect on CRAE. For example, a 1 mmol/L increase in TG was associated with a narrowing of 1.33 µm (*p* = 0.031). Adolescents in the highest quartile of TGs (≥2.0 mmol/L) had a CRAE narrowing of 3.2 µm compared to adolescents in the lowest quartile (<1.0 mmol/L) [17].

There was also a strong association of TC with narrower CRAE, where for every 1 mmol/L increase in TC, there was a CRAE reduction of 1.83 µm (*p* < 0.01). Those participants who had TC levels > 5.5 mmol/L had a CRAE narrowing of 4.1 µm when compared to those with TC levels < 4.0 mmol/L [17]. Additionally, TC was negatively associated with SCP vessel density in multiple retinal regions (all *p* < 0.05) [18].

The effect of LDL-C was more clear and demonstrated an effect through dose dependence. Every 1 mmol/L increase in LDL-C resulted in a CRAE reduction of 1.92 µm (*p* = 0.006). Adolescent participants in the highest LDL-C quartile (≥4.0 mmol/L) on average had a CRAE narrowing of 5.2 µm [17]. LDL-C was also negatively associated with SCP vessel density (although not statistically significant (*p* = 0.107)) [18].

Of all the lipid parameters, apoB had the greatest effect on CRAE [17], with participants in the highest apoB quartile having a CRAE decrease of −7.18 µm compared to the lowest apoB quartile (*p* = 0.009) [17].

HDL-C was positively associated with vessel density in the RPC (*p* ≤ 0.010), where participants with increased HDL-C had higher RPC vessel density. No significant association was found with CRAE [18].

CRVE was not significantly associated with lipid parameters (all *p* > 0.10) [17]. A summary of the lipid parameters and their effect on CRAE, CRVE, and RPC vessel density is presented in Table 2.

### 3.2. Retinal Microvascular Alterations in Familial Hypercholesterolemia

FH patients had significantly larger FAZ versus healthy controls (0.341 ± 0.111 mm^2^ vs. 0.275 ± 0.07 mm^2^; *p* = 0.031). The SCP vessel density was significantly lower in FH patients compared to healthy controls in the total retina (49.59 ± 2.39% vs. 51.45 ± 2.03; *p* = 0.018), the parafoveal area (51.22 ± 2.39% vs. 54.03 ± 2.43; *p* = 0.016), and the perifoveal area (50.51 ± 2.33% vs. 52.26 ± 2.14; *p* = 0.014). There were significantly higher DCP vessel density values in respectively in some subsectors (all *p* < 0.05). Homozygous FH (HoFH) patients had even lower SCP density, especially in the parafoveal and perifoveal areas, when compared to healthy controls. Higher DCP perifoveal vessel density was associated with more severe cardiovascular symptoms in FH patients (OR 2.67, 95% CI 0.33–5.01, *p* = 0.025) [18]. The main differences in retinal microvascular parameters between patients with FH and healthy controls are listed in Table 3.

### 3.3. Sex-Specific Trends

In terms of retinal vessel calibers, there were substantial sex differences. Boys had lower CRAE values than girls across all age categories. In children aged 7–11 years, CRAE was 149.6 ± 13.7 µm for boys and 153.4 ± 12.8 µm for girls (*p* = 0.014). In adolescents (12–19 years), CRAE was 146.1 ± 14.0 µm for boys compared with 150.9 ± 12.1 µm for girls (*p* < 0.001). A similar difference was also seen for CRVE, with the younger boys having CRVE of 216.1 ± 17.6 µm, while girls had wider venular diameters at 221.7 ± 18.3 µm (*p* = 0.009). The difference was also present in adolescents, where boys’ CRVE was 213.1 ± 20.9 µm while girls’ CRVE was 217.5 ± 18.7 µm (*p* = 0.019) [15]. In another large cohort study, there were similar sex differences where girls had considerably wider CRAE (211.3 ± 15.4 µm) and CRVE (239.0 ± 16.6 µm) compared with boys, with CRAE (204.5 ± 15.2 µm) and CRVE (233.4 ± 15.3 µm), although there was no significant difference between sexes for arteriolar-to-venular ratio (AVR; *p* < 0.001) [16].

The mean arterial blood pressure (MAP) was significantly higher in boys than in girls (at least in adolescence). The boys had a mean MAP of 80.2 ± 9.05 mmHg, and the girls’ MAP was 77.9 ± 9.33 mmHg (*p* = 0.005) [15]. Other research has also shown a difference between sexes in MAP in another large pediatric cohort study where boys had an MAP of 85.6 ± 7.9 mmHg, while girls had an MAP of 83.7 ± 7.5 mmHg (*p* < 0.001) [16].

Boys also had higher rates of dyslipidemia as indicated by higher LDL-C and TG. As an example, in adolescents, 27% of the boys had LDL-C levels ≥ 3.0 mmol/L compared to girls (with similar rates at 18%, *p* < 0.05). Rates of TG levels ≥ 1.7 mmol/L also followed the same pattern, with boys showing 22% versus 15% in girls (*p* = 0.02). Boys experienced a significantly larger impact of dyslipidemia on their CRAE by 4.8 µm compared to girls and were similarly impacted at 3.2 µm (*p* = 0.03) [17].

A summary of measured differences is offered in Table 4, including differences in retinal vessel diameters, blood pressure, and dyslipidemia.

### 3.4. Combined Effects of Obesity and Dyslipidemia

Adolescents with dyslipidemia and obesity experienced the greatest arteriolar narrowing with a resulting CRAE reduction of 4.8 µm, compared with a 2.5 µm reduction for dyslipidemia alone and a 2.1 µm reduction for obesity alone (*p* < 0.001). While CRVE was not significantly impacted by either condition alone, a cumulative effect existed with an overall reduction of 2.9 µm (*p* = 0.04). Coexistence of both conditions existed in 15% of adolescents and occurred significantly more frequently in urban than rural (18% vs. 10%, *p* = 0.02) areas [17].

### 3.5. Body Composition and Retinal Microvascular Calibers

Higher fat mass (FM) demonstrated a strong relation to narrower CRAE in adolescents aged 12–19 years. Specifically, CRAE narrowed by 0.47 µm for a 1 kg increase in FM (*p* < 0.05). Participants in the highest and lowest FM quartiles had a CRAE difference of 3.8 µm between individuals who were in the highest FM quartile (≥15 kg compared to the lowest FM quartile (<7 kg); *p* < 0.01) [15].

In contrast, fat-free mass (FFM) showed a significant positive relation to CRVE, with an increase for every 1 kg increase in FFM of 0.25 µm (*p* = 0.04). This was higher for adolescents with FFM > 40 kg [15].

Increased body water percentage (BWP) at the 1% level corresponded to a 0.33 µm widening of CRAE and a 0.64 µm narrowing of CRVE (both *p* < 0.001). Adolescents with BWP above 65% had significantly wider CRAE and smaller CRVE than those who did not have the same hydration levels [15].

Overweight and obese children had wider CRVE than normal-weight peers, with an average difference of 6.2 µm (*p* = 0.03). AVR was lower in overweight (*p* = 0.048) and obese participants (*p* = 0.038). BMI was negatively related to CRAE (β = −0.74 µm per kg/m^2^, *p* = 0.04) and positively related to CRVE (β = +0.82 µm per kg/m^2^, *p* = 0.02). Physical inactivity was negatively associated with AVR (β = −0.013, *p* = 0.025) [16].

The individual effects of FM, FFM, BWP, BMI, and obesity on CRAE and CRVE are displayed in Table 5.

## 4. Discussion

This systematic review examined the effects of specific lipid levels (TG, TC, LDL-C, HDL-C, and apoB) and body composition variables (FM, FFM, and BWP) on retinal microvascular structure in children and adolescents, focusing on arteriolar narrowing and venular changes. There appears to be a possible relationship between elevated lipid levels, negative body composition measurements, and retinal vascular measurement, especially CRAE narrowing [15,17], even in children who are at a healthy weight, as there is evidence to indicate that abnormal cholesterol can exist regardless of BMI [19]. This is consistent with previous reports that dyslipidemia can lead to this same effect in adults due to microvascular endothelial dysfunction and increased cardiovascular risk [20,21]. However, the fact that we found these changes in adolescents establishes early identification and treatment of dyslipidemia as a priority so that there may be no long-term vascular complications.

We found evidence for higher FM being associated with CRAE narrowing [15], which is consistent with evidence that adiposity contributes to systemic inflammation, oxidative stress, and endothelial dysfunction that all ultimately lead to an increased vascular resistance and disruption in the regulation of blood flow [22]. Interestingly, FFM was associated with widening of CRVE [15], which aligns with research studying lean body mass and metabolic demand along with venular structure [23]. Lastly, BWP was found to protect both CRAE and CRVE [15], to recognize the role that hydration status plays in being able to maintain vascularity and prevent microvascular disruption [24]. It seems these relationships are age dependent [15], and the effect appears to apply to various dimensions of body composition. For example, BMI, a marker of body composition, has been demonstrated to negatively correlate with CRAE and positively correlate with CRVE for children [16]. Similarly, greater BMI and hip circumference have been associated with greater carotid intima-media thickness (cIMT), which again represents support for the early vascular burden of adiposity across micro and macrovascular beds [25]. Dyslipidemia, particularly elevated TG, TC, LDL-C and apoB, was significantly related to CRAE narrowing, with apoB demonstrating the strongest dose-dependent effect [17], further reinforcing its clinical utility as an early vascular risk indicator in children.

Importantly, while there was no statistical association between lipid parameters and CRAE in the unadjusted models, these relationships did become statistically significant after adjusting for other variables, including axial length, BMI, MAP, and fellow eye vascular caliber [17]. This indicates that some of the relationship between lipid levels and CRAE is likely being confounded or mediated by a number of anthropometric and hemodynamic factors. Therefore, the associations found in this study should be viewed as conditional, rather than as an independent effect of lipid exposure.

In addition, the lipid measurements used in one of the major pediatric cohorts were taken while the children were not fasting [17], which could result in variability in the lipid levels measured, particularly in triglycerides, and could influence the strength of the associations observed.

It should also be noted that there were substantial differences in the absolute CRAE measurements among the different cohorts studied.

For example, the mean CRAE ranged from approximately 146–150 µm in two Chinese cohorts [17] to greater than 200 µm in a single European cohort [16]. These large differences in CRAE values cannot be attributed entirely to lipid exposure and likely reflect methodological differences in how CRAE was measured among the cohorts, including the type of camera used, image resolution, measurement zone of the CRAE about the optic disk, software algorithm (e.g., the Knudtson formulas [13]), whether adjustments were made for axial length or refractive error, ethnic variations in retinal vascular phenotypes and differences in age and blood pressure distributions among the cohorts. As such, comparisons of absolute CRAE values across studies should be viewed with caution, with a focus on the associations within each study. Notably, within the included cohorts, adolescents with dyslipidemia consistently exhibited narrower retinal arteriolar calibers than their normolipidemic peers, supporting an association between adverse lipid profiles and early retinal microvascular alterations [17].

Dyslipidemia was noted not to have any significant association with CRVE in children [17], which contrasts with adult studies where both arteriolar and venular changes are typically observed [4]. One potential explanation may be that microvascular arteriolar dysfunction evolves earlier in life, with venular change occurring later as a result of lipid exposure. Additionally, HDL-C was positively related to vessel density in the RPC [18], highlighting its potential protective role in microcirculatory function.

Additional evidence of the relatedness of dyslipidemia can be seen in children with FH who presented larger FAZ and reduced SCP vessel density in the parafoveal and perifoveal regions [18]. These findings could indicate early microvascular ischemia and endothelial dysfunction. Similarly, FH children’s increased DCP vessel density may represent a compensatory change for impairment at the level of the superficial capillary. The severity of these changes correlated with the degree of dyslipidemia [18], supporting a dose-dependent relationship between lipid abnormalities and retinal microvascular changes.

In addition, there were sex-related differences where boys had narrower CRAE and CRVE than girls, especially during adolescence [15], which again supports previous evidence that suggests hormonal differences in lipid metabolism, vascular tone, and endothelial responsiveness contribute to sex-specific vascular risk [26]. Notably, boys with dyslipidemia had more marked CRAE narrowing than girls with similar lipid profiles [16,17], again supporting prior reports where the vascular system of boys is more susceptible to lipid-related endothelial damage [26].

Perhaps not surprisingly, the greatest CRAE narrowing was attributed to the combination of obesity and dyslipidemia [17], which supports some preliminary concepts that metabolic stressors interact in a synergistic way to exacerbate microvascular dysfunction [27]. The available studies did not evaluate the underlying causes of obesity; therefore, it remains unclear whether the observed retinal microvascular changes were attributable to dyslipidemia alone or to the combined influence of obesity and other metabolic and lifestyle factors. While neither obesity nor dyslipidemia was shown to significantly affect CRVE, a marked reduction was evident in adolescents with both conditions [17], suggesting venular remodeling may reflect a more advanced state of metabolic disruption regardless of underlying health state.

This study’s data provide preliminary evidence that retinal imaging may have the potential to identify early vascular consequences of dyslipidemia in youth; however, it is very preliminary and limited due to the low number of current studies. Recent recommendations for universal lipid screening, particularly for FH, demonstrate the need for early identification [28,29]. Given its noninvasive nature and the established link between FH and premature atherosclerosis [28,29], retinal imaging could in the future contribute to identifying at-risk individuals, but this role requires confirmation in larger longitudinal cohorts.

### Strengths and Limitations

In this review, CRAE/CRVE were obtained from fundus photography in the included studies, while OCT-A metrics were available only from one preprint study. The pediatric nature of the review and objective measures of the blood vessels have led us to suggest that the retina could be an early indicator of metabolic risk.

The preponderance of Chinese pediatric cohorts limits both the applicability of results to other ethnic populations as well as the generalizability of these findings across different health care environments. Of the three, two of them [15,17] come from the same cohort (Guangzhou Twin Eye Study)—while there could potentially be some degree of participant overlap, which does not significantly affect the narrative synthesis as a whole. Although narrative synthesis minimizes the risk of statistical double-counting, shared cohort characteristics may influence the apparent consistency of findings. In addition, one study [18], which contains the OCT-A data, is a preprint. As such, caution is needed when interpreting the results of this study. Furthermore, OCT-A findings were derived from a single eligible study and should therefore be interpreted as preliminary evidence requiring confirmation in additional pediatric cohorts.

However, our ability to compare and infer causality is limited due to a small number of studies, differences in methodology, and the predominance of cross-sectional designs. The major limitation of this review is the very small number of eligible studies (*n* = 4), with heterogeneous methodologies and populations, which restricts the strength and generalizability of conclusions. Potential confounders, variability in study age groups, generalizability, and the lack of interventional studies prevent us from concluding and exercising reversibility. In addition, information regarding comorbid conditions such as hypertension and diabetes mellitus was limited or not consistently reported across the included studies, restricting further assessment of their potential influence on retinal microvascular findings. Moreover, the magnitude of CRAE changes associated with lipid parameters was modest (generally 1–2 µm per mmol/L), and the clinical relevance of such small caliber differences remains uncertain. Pediatric OCT-A data also remain scarce, limiting conclusions regarding retinal microvascular perfusion parameters. These limitations highlight important gaps in the current literature, particularly the lack of longitudinal studies evaluating the progression of retinal microvascular changes over time and interventional studies assessing the effects of lipid-lowering or lifestyle interventions. Future longitudinal studies with standardized retinal imaging protocols would help clarify the clinical significance of these findings and their long-term implications.

## 5. Conclusions

Retinal microvascular changes, particularly CRAE narrowing, are consistently linked with dyslipidemia and less favorable body composition measures in children and adolescents. These early changes are more pronounced in boys and those with obesity in conjunction with dyslipidemia. Consequently, these findings underline the retina as a potentially useful biomarker of early vascular dysfunction, as they are non-invasive. Retinal imaging can assist in identifying youth at risk who might otherwise go unnoticed by a healthcare provider. However, given the limited number of studies and their methodological heterogeneity, the clinical significance of these findings remains uncertain.

## Figures and Tables

**Table 1:** Eligibility criteria for inclusion of studies.

Criteria	Inclusion Criteria	Exclusion Criteria
Study Focus	Studies on dyslipidemia and retinal microvascular abnormalities, including epidemiology, detection, and treatment.	Studies not focused on dyslipidemia or retinal microvascular health.
Study Design	RCTs, cohort studies, case–control studies, cross-sectional studies.	Review articles, editorials, case reports, or studies lacking sufficient methodological detail.
Language	Only studies published in English.	Studies published in languages other than English.
Lipid Profile Data	Studies providing data on TC, LDL-C, HDL-C, and TG.	Studies without detailed lipid profile data.
Body Composition Assessment	Studies incorporating BMI, BIA, or DEXA.	Studies without body composition assessments.
Population	Studies involving children and adolescents.	Studies conducted exclusively on adults.
Animal/Human Studies	Studies conducted exclusively on human participants.	Studies involving animal models.

Abbreviations: RCTs, randomized controlled trials; TC, total cholesterol; LDL-C, low-density lipoprotein cholesterol; HDL-C, high-density lipoprotein cholesterol; TG, triglycerides; BMI, body mass index; BIA, bioelectrical impedance analysis; DEXA, dual-energy X-ray absorptiometry.

**Table 2:** Associations Between lipid parameters and retinal microvascular measures (CRAE, CRVE, RPC).

Lipid Profile	CRAE Change (µm)	CRVE Change (µm)	RPC Change
TG	−1.33 µm/mmol/L	No significant change	No significant change
TC	−1.83 µm/mmol/L	No significant change	No significant change
LDL-C	−1.92 µm/mmol/L	No significant change	No significant change
ApoB	−7.18 µm (quartile)	No significant change	No significant change
HDL-C	No significant change	No significant change	Increased vessel density (*p* ≤ 0.010)

Abbreviations: CRAE, central retinal arteriolar equivalent; CRVE, central retinal venular equivalent; RPC, radial peripapillary capillaries; TG, triglycerides; TC, total cholesterol; LDL-C, low-density lipoprotein cholesterol; HDL-C, high-density lipoprotein cholesterol; ApoB, apolipoprotein B.

**Table 3:** Retinal microvascular parameters in familial hypercholesterolemia vs. controls.

Retinal Parameter	FH Patients (Mean ± SD)	Healthy Controls (Mean ± SD)	*p*-Value
FAZ (mm^2^)	0.341 ± 0.111	0.275 ± 0.070	0.031
SCP—Whole Retina (%)	49.59 ± 2.39	51.45 ± 2.03	0.018
SCP—Parafoveal (%)	51.22 ± 2.39	54.03 ± 2.43	0.016
SCP—Perifoveal (%)	50.51 ± 2.33	52.26 ± 2.14	0.014
DCP—Selected Sectors	Increased	—	*p* < 0.05
DCP–Perifoveal (CV symptoms)	Increased in patients with severe CV symptoms	—	0.025 (OR 2.67, 95% CI 0.33–5.01)

Abbreviations: FAZ, foveal avascular zone; SCP, superficial capillary plexus; DCP, deep capillary plexus; CV, cardiovascular; OR, odds ratio; CI, confidence interval.

**Table 4:** Sex-based differences in retinal vascular caliber, blood pressure, and lipid profiles.

Measurement	Boys	Girls	*p*-Value
CRAE (7–11 years)	149.6 µm	153.4 µm	*p* = 0.014
CRAE (12–19 years)	146.1 µm	150.9 µm	*p* < 0.001
CRVE (7–11 years)	216.1 µm	221.7 µm	*p* = 0.009
CRVE (12–19 years)	213.1 µm	217.5 µm	*p* = 0.019
MAP	80.2–85.6 mmHg	77.9–83.7 mmHg	*p* < 0.001
LDL-C ≥ 3.0 mmol/L Prevalence	27%	18%	*p* < 0.05
TG ≥ 1.7 mmol/L Prevalence	22%	15%	*p* = 0.02
CRAE reduction in dyslipidemia	4.8 µm	3.2 µm	*p* = 0.03

Abbreviations: CRAE, central retinal arteriolar equivalent; CRVE, central retinal venular equivalent; MAP, mean arterial pressure; LDL-C, low-density lipoprotein cholesterol; TG, triglycerides.

**Table 5:** Effects of body composition parameters on CRAE and CRVE.

Body Composition Parameter	CRAE Change (µm)	CRVE Change (µm)
FM	−0.47 µm/kg	+0.25 µm/kg
FFM	Not significant	+0.25 µm/kg
BWP	+0.33 µm/unit	−0.64 µm/unit
BMI	−0.74 µm per kg/m^2^	+0.82 µm per kg/m^2^
Overweight/Obesity	Not reported	+6.2 µm vs. normal weight

Abbreviations: CRAE, central retinal arteriolar equivalent; CRVE, central retinal venular equivalent; FM, fat mass; FFM, fat-free mass; BWP, body water percentage; BMI, body mass index.

## Data Availability

No new data were created or analyzed in this study.

## Supplementary Materials

The following supporting information can be downloaded at: https://www.mdpi.com/article/10.3390/children13060824/s1, Table S1: Detailed search strategy used for database searches; Table S2: Risk of bias assessment of included studies using the Newcastle–Ottawa Scale (NOS).

## Abbreviations

The following abbreviations are used in this manuscript:

FH	Familial Hypercholesterolemia
PRISMA	Preferred Reporting Items for Systematic Reviews and Meta-Analyses
LDL-C	Low-Density Lipoprotein Cholesterol
OCT-A	Optical Coherence Tomography-Angiography
CRAE	Central Retinal Arteriolar Equivalent
CRVE	Central Retinal Venular Equivalent
BIA	Bioelectrical Impedance Analysis
TG	Triglycerides
TC	Total Cholesterol
Apol-B	Apolipoprotein B
HoFH	Homozygous Familial Hypercholesterolemia
NOS	Newcastle-Ottawa Scale
RCTs	Randomized controlled trials
BMI	Body mass index
RPC	Radial Peripapillary Capillaries
FAZ	Foveal Avascular Zone
SCP	Superficial Capillary Plexus
DCP	Deep Capillary Plexus
HDL-C	High-Density Lipoprotein Cholesterol
MAP	Mean Arterial Blood Pressure
FM	Fat Mass
FMM	Fat-Free Mass
BWP	Body Water Percent
