# Supplementary material for: Dyslipidemia and Retinal Microvascular Health in Children and Adolescents: A Systematic Review
Source: Children (Basel). 2026 Jun 17;13(6):824. doi: 10.3390/children13060824 (PMC13298365; doi:10.3390/children13060824)
Supplement: Supplementary file 1 [file children-13-00824-s001.zip › children-4360532-supplementary.pdf]

Supplementary Material

Supplementary Table S1. Detailed search strategy used for database searches

Databases searched: PubMed, Embase, and the Cochrane Library

Date of last search: December 2024

PubMed search strategy:

("Dyslipidemias"[Mesh] OR dyslipidemia OR "Body Composition"[Mesh] OR "body composition" OR "Familial Hypercholesterolemia"[Mesh] OR "familial hypercholesterolemia")

AND

("Retinal Microvasculature"[Mesh] OR "retinal microvasculature" OR "microvascular changes" OR CRAE OR CRVE OR "Optical Coherence Tomography Angiography" OR OCT-A)

Embase search strategy:

('dyslipidemia'/exp OR 'body composition'/exp OR 'familial hypercholesterolemia'/exp)

AND

('retinal microvasculature'/exp OR 'microvascular changes' OR CRAE OR CRVE OR 'optical coherence tomography angiography'/exp)

Cochrane Library search strategy:

(dyslipidemia OR "body composition" OR "familial hypercholesterolemia")

AND

("retinal microvasculature" OR "microvascular changes" OR CRAE OR CRVE OR OCT-A)

Limits applied:

- Human studies
- English language
- Pediatric population (children and adolescents)

Supplementary Table S2. Risk of bias assessment of included studies using the Newcastle–Ottawa Scale (NOS)

Study	Selection (0–4)	Comparability (0–2)	Outcome/Exposure (0–3)	Total Score	Quality
Xiao et al., 2015 [15]	4	1	2	7	High
Hanssen et al., 2012 [16]	4	1	2	7	High
Xiao et al., 2017[17]	4	2	2	8	High
Wang et al., 2025 [18]	3	1	2	6	Moderate

NOS scores range from 0 to 9. Studies scoring ≥7 points were considered high quality, while scores of 5–6 points indicated moderate quality.
